# Aotearoa New Zealand pet guardians’ attitudes towards the financial cost of pet care

**DOI:** 10.1017/awf.2025.10048

**Published:** 2025-11-24

**Authors:** Leena Awawdeh, Natalie Waran, Rachel H. Forrest

**Affiliations:** 1 School of Agricultural, Environmental and Veterinary Sciences, Charles Sturt University - Wagga, Wagga, NSW, Australia; 2 School of Nursing (Te Kura Tāpuhi), College of Health (Te Kura Hauora Tangata), Massey University (Te Kunenga ki Pūrehuroa), Manawatū, New Zealand; 3 Navigate Animal Welfare, Hawke’s Bay, Napier, New Zealand; 4 Faculty of Education, Humanities and Health Science, Eastern Institute of Technology, Hawke’s Bay, New Zealand

**Keywords:** Animal welfare, companion animals, financial impact, pet care, pet health insurance, veterinary costs

## Abstract

Pet guardianship is a common practice globally that enhances human well-being by offering companionship and emotional support. However, it also entails financial responsibilities that can pose challenges to both human and animal well-being. This study used an online survey distributed between January and March 2019 to adults residing in Aotearoa New Zealand (NZ), to investigate financial aspects of pet guardianship, including the importance of pet insurance, the impact of veterinary costs, and the ethical considerations of owning a pet. Understanding these aspects is crucial for promoting responsible pet ownership and ensuring animal welfare. Data were collected through a nationwide online survey, part of the Furry Whānau Wellbeing research project. The survey included questions regarding the financial aspects of pet guardianship, and respondents were asked to indicate their level of agreement with various statements. A total of 2,744 respondents participated in the survey. Of these, 2,358 respondents answered the pet guardianship section. Among them, 885 (37.5%) owned both cats and dogs, 652 (28%) were cat-only owners, 609 (26%) were dog-only owners, and 212 (9%) did not currently own a cat or dog. The cost of veterinary care was identified as a key concern, with 1,924 out of 2,296 (83.9%) respondents agreeing that the expense affects the level of healthcare their pets receive. Only one-third of respondents (825/2,296) considered pet insurance essential due to high costs and exclusions. The study also revealed disparities for rural and low-income households. The financial well-being of pet guardians presents a complex challenge, affecting both the well-being of the pet and its owner. This research highlights the need for comprehensive strategies to promote sustainable and equitable pet guardianship, including improving access to affordable veterinary care, enhancing financial literacy among pet owners, and providing support systems for those facing economic hardship.

## Introduction

Recent global studies reinforce the notion that financial difficulties can significantly impact both the quality and timeliness of veterinary care for companion animals, particularly among financially disadvantaged households (King *et al.* 2022; Muldoon & Williams 2024a,b). Preventive care, such as vaccination, desexing, dental procedures, and parasitic control, is often delayed or forgone due to cost, causing health to deteriorate and, later, requiring more expensive interventions (Morris *et al.* 2021).

The COVID-19 epidemic put further pressure on household budgets and worsened disparities as regards access to veterinary services, especially in rural and disadvantaged areas (Applebaum *et al.* 2020; Bibbo *et al.* 2022). The wider implications of the issue are increasingly becoming recognised, particularly through a welfare structure lens, which emphasises the mutual relations between animal welfare, human welfare and socioeconomic conditions (Protopopova 2021). When pet guardians are unable to provide essential care, the emotional toll can contribute to stress and feelings of moral failure, which can affect animal welfare and the overall well-being of human families (Morris *et al.* 2021). Despite an increasing focus on these concerns internationally, there has been a paucity of studies examining these dynamics in Aotearoa New Zealand (NZ). Previous studies have noted concerns regarding the uptake and value of pet insurance (Gates *et al.* 2019; Forrest *et al.* 2023), which can influence decisions about the financial stress of caring for companion animals.

Globally, pet guardianship is a widespread practice, with companion animals playing an integral role in the lives of millions of people (Wood *et al.* 2017). The human-animal bond brings many benefits, including companionship, emotional support, and improved physical and mental well-being (Fraser *et al.* 2020). However, alongside these positive aspects, pet guardianship also entails financial responsibilities that can pose challenges for many individuals and families. The costs associated with pet care, encompassing routine veterinary visits, vaccinations, preventive medications, and potential emergency treatments, can accumulate significantly over time (Gates *et al.* 2019). These financial obligations can be particularly burdensome for low-income households, possibly leading to complicated decisions regarding their pets’ health and well-being (Applebaum *et al.* 2020). Even people with stable incomes can find their finances strained by unexpected veterinary bills or specialised care.

Furthermore, the cost of veterinary care for pets is increasing due to advancements in small animal practice, including specialisation, complex diagnostics, and advanced treatments (Brown *et al.* 2021). These increased costs are a major obstacle to accessing care for many animal guardians (Kipperman *et al.* 2017; Benson & Tincher 2024). The increasing complexity of veterinary medicine also introduces ethical challenges, such as balancing patient interests with financial constraints and client expectations (Kipperman *et al.* 2017; Springer *et al.* 2019; Quain *et al.* 2021). Veterinarians report that economic limitations affect their ability to ensure optimal care and contribute to burn-out (Kipperman *et al.* 2017). Strategies to address these issues, such as improving customer education, discussing costs and pet insurance with customers, and implementing various care approaches, are recommended. Despite these challenges, better patient care and timely interventions (Ackerman 2024) continue to enhance veterinarians’ ability to improve outcomes.

Pet health insurance offers several advantages, including an increased willingness to pay for veterinary care and a reduced likelihood of pre-surgical euthanasia (Anderson *et al.* 2021; Chiu *et al.* 2021). Insured pet guardians tend to spend more on veterinary services, potentially improving access to care (Williams *et al.* 2020). However, pet insurance has limitations, particularly for owners with limited financial resources who struggle to afford premiums or veterinary costs (Becker *et al.* 2022). The uptake of pet insurance remains relatively low, with only a minority of pet owners utilising it (Chiu *et al.* 2021). Factors influencing insurance adoption include education about treatment costs and disease risks (Chiu *et al.* 2021). While pet insurance can reduce the costs associated with veterinary clinics (Becker *et al.* 2022), its uptake has been slow (Paul & Skiba 2012).

Understanding the attitudes and experiences of pet guardians regarding the financial aspects of having a pet is crucial for promoting responsible pet care and ensuring the welfare of companion animals. Studies show that pet guardians generally report positive experiences, with women exhibiting higher bonding levels (Meier & Maurer 2022). However, financial costs represent a significant concern, especially for older adults with limited resources (Lian & Mathis 2016; Meier & Maurer 2022). Community-based veterinary programmes can help address these financial barriers (Kogan *et al.* 2021). Communication between veterinarians and pet guardians regarding costs remains a challenge, with differing perspectives on the value of services (Coe *et al.* 2007). Attitudes towards responsible pet guardianship vary based on gender and guardian status (Selby *et al.* 1979). Overall, promoting pet guardianship while addressing financial and practical concerns could enhance the well-being of both humans and animals (Anderson *et al.* 2015; Fox & Gee 2019).

While Aotearoa NZ pet guardians generally recognise the importance of proper care practices, there are knowledge gaps, particularly around the need for regular veterinary visits and the impact of breeding on animal welfare (Gates *et al.* 2019; Forrest *et al.* 2023). Also, studies have found that while pet guardians value the mental, physical, and social benefits of having a pet, the high costs of veterinary care and pet insurance are major concerns (Abra *et al.* 2019; Byard & Langlois 2020). Financial constraints have also been identified among NZ pet guardians, particularly for lower-income households, which often lead to compromised care or difficult decisions regarding their pets (Forrest *et al.* 2023).

Internationally, studies have shown similar trends. For instance, Abra *et al.* (2019) and Byard and Langlois (2020) reported that pet guardians value the mental, physical, and social benefits of pet ownership but are often burdened by the rising costs of veterinary care and insurance. Muldoon and Williams (2024b) further noted that financial constraints can compromise animal welfare and place emotional strain on pet owners in economically vulnerable households. Despite the importance of these issues, limited research exists on the attitudes of NZ pet guardians regarding financial responsibilities towards their pets. To address this knowledge gap, part of the Furry Whānau Wellbeing research study, funded by the NZ Companion Animal Trust (NZCAT), explored pet owners’ financial responsibilities, including the importance of pet insurance, the impact of veterinary costs, and the ethical considerations of owning a pet (Forrest *et al.* 2019). By shedding light on these multifaceted issues, this research explores methods for enhancing our communities’ understanding of their responsibilities as pet owners so we can implement effective behaviour change programmes to benefit the welfare of companion animals (dogs and cats) in NZ.

Furthermore, research on the financial aspects of pet guardianship not only addresses animal welfare and public health issues but also has the potential to impact the global targets established by the United Nations Sustainable Development Goals (SDGs). Specifically, this study contributes to SDG 3 (Good Health and Well-being), SDG 10 (Reduced Inequalities) and SDG 11 (Sustainable Cities and Communities), highlighting the importance of equitable access to veterinary care as an aspect of holistic well-being.

## Materials and methods

### Ethical statement

This study was conducted in accordance with the guidelines of the Declaration of Helsinki and was approved by the Eastern Institute of Technology Research and Ethics Approval Committee (REAC, ref: 19/53) and informed consent was obtained from all subjects involved in the study.

### Data collection

Data for this study were collected as part of the 2019 Furry Whānau Wellbeing research project funded by the NZCAT (Eastern Institute of Technology’s Research and Ethics Approval Committee ref: 19/53). Detailed information regarding participants, survey questions, and data collection methods can be found in Forrest *et al.* (2019). In short, a nationwide online survey was open to NZ pet guardians aged 18 and over from January 8 to March 31, 2019. This study focuses on an in-depth analysis of the responses to Question 38 of the survey, which specifically addressed the economic impact of pet guardianship. The question read as follows: Please choose the option (Strongly agree, Agree, Neutral, Disagree, Strongly disagree) that most closely describes how you feel about the following statements: ‘Expense is a factor in the level of vet/animal healthcare that dogs and/or cats receive’; ‘If my income were higher, I would spend more money on my dog and/or cat’s health and well-being’; ‘Pet insurance is important’; ‘People should only own a pet if they can afford to keep it’; ‘Owning a pet has caused me financial stress (vet bills)’. The data presented in this study are available upon request from either of the corresponding authors (LA or RF).

### Statistical analysis

Respondents had the option to skip any questions or sub-questions in the survey. The quantitative data, which consisted of forced responses to survey questions, were analysed using descriptive statistics. For each statement, cross-tabulations and Chi-squared and z-tests (α = 0.05, with Bonferroni corrections) were used to explore whether the respondents’ choices were associated with gender (female, male), ethnicity (Māori, NZ European, Other), age (18–24 years, 25–34 years, 35–44 years, 45–54 years, 55–64 years, 65–74 years, 75–84 years), income range (< NZ$14,000, NZ$14,000–NZ$48,000, NZ$48,000–NZ$70,000, NZ$70,000–NZ$100,000, > NZ$100,000, would rather not say), qualification level (1–10), whether they had a child or children (yes, no), whether they had a rural upbringing (yes no), or whether they were currently living in a town/city (yes, no).

To identify the most significant predictors of agreement for each statement, a forward stepwise binary logistic regression based on the likelihood-ratio test was employed. Three separate analyses were performed for the following dependent variables: (1) ‘Expense is a factor in the level of vet/animal health care that dogs and/or cats receive’; (2) ‘Pet insurance is important’; and (3) ‘People should only own a pet if they can afford to keep it’. For each variable, responses were coded as 1 for agreement (which included agree and strongly agree responses) and 0 for did not agree (which included neutral, disagree and strongly disagree responses). The probability for variable entry was set at *P* < 0.05, and for removal at *P* > 0.10. The overall significance of each final model was evaluated using the Omnibus Test of Model Coefficients, and the proportion of variance explained was estimated using the Nagelkerke R^2^ statistic. Odds ratios were calculated to interpret the effect of each significant predictor. All statistical analyses were carried out using IBM SPSS® Statistics software (version 25). The qualitative data were derived from open-ended responses to Question 38, where participants were invited to describe how the financial cost of care affected their ability to support their pets. Two researchers analysed the qualitative data independently for emergent themes and categories using a general inductive approach (Thomas 2006) to minimise the likelihood of researcher bias.

## Results

### Demographic description of the respondents

A total of 2,744 individuals across NZ responded to the online survey. A detailed demographic breakdown of all respondents, including cat and dog owners, is provided in our previous publications (Forrest *et al.* 2019, 2022, 2023; Awawdeh & Forrest 2024). The sample was predominantly female (92.3%), with males significantly underrepresented at 7.7%, compared to 49.4% in the 2018 NZ census. Māori were also underrepresented, comprising 8.3% of respondents compared to 16.5% nationally. Most respondents identified as NZ European (83.4%), followed by Other European (10.1%), with smaller proportions identifying as Māori, Pacific Peoples, Asian, or other ethnicities. Respondents represented all age ranges from 18 to 85+, with Māori present across all age groups except for those aged 85 and older.

Household income data were provided by 2,251 respondents and were relatively evenly distributed across income brackets above NZ$14,000. There was a noticeably lower representation of respondents reporting household incomes below NZ$14,000. Across ethnic groups, Māori and NZ European respondents were similarly represented across income brackets, although some regional and demographic variability was observed. Weak but statistically significant associations were found between income and the number of adults and children in the household. Of the 2,358 respondents who answered the pet guardian questions, 37.5% (n = 885) owned both types of pets, 28% (n = 652) were cat-only owners, and 26% (n = 609) were dog-only owners. Nine per cent (n = 212) of the respondents owned neither a cat nor a dog at the time of completing the survey but had been pet owners previously.

A total of 2,298 respondents answered Q38 and indicated their level of agreement with statements regarding the financial costs associated with pet care. The demographics for this subsample were similar to those of the entire sample, indicating there was no obvious selection bias associated with a particular demographic when choosing to answer (or skip) this question.

For each statement, there were between 2,287 and 2,296 responses. Table 1 shows that more than 80% of respondents agreed or strongly agreed that expense is a factor in the level of healthcare that dogs and/or cats receive, and that people should only own a pet if they can afford to keep it. Only 36% of respondents considered pet insurance to be important.

**Table 1.** Percentages of 2019 New Zealand Pet Survey respondents selecting each level of agreement for each statement regarding certain financial aspects of pet care

	Strongly agree	Agree	Total Agreeing	Neutral	Disagree	Strongly disagree	Total Disagreeing
Expense is a factor in the level of vet/animal health care that dogs and/or cats receive (n = 2,293)	37.8%	46.1%	83.9%	6.2%	6.1%	3.8%	10.0%
Pet insurance is important (n = 2,287)	14.3%	21.6%	35.9%	46.7%	14.3%	3.2%	17.4%
People should only own a pet if they can afford to keep it (n = 2,296)	56.8%	32.1%	88.8%	8.1%	2.2%	0.8%	3.1%

A total of 319 respondents left comments, and several themes emerged (see Table 3). The major themes were that veterinary care and pet insurance are expensive and that pet insurance often has exclusions; that pets should only be owned by those who can afford them and that pets are a luxury and a privilege; that if you own a pet, you need to save for unexpected costs and that sometimes situations change and that there should be financial assistance; that pet care needs to be more affordable; that guardians have differing priorities; that sometimes costs result in pets being euthanased and that education and planning are important.

For the statement ‘Expense is a factor in the level of vet/animal health care that dogs and/or cats receive’, household income level was associated with a higher percentage of those respondents with a household income between NZ$48,000–NZ$70,000 selecting strongly agree (45 versus 33%) and a lower percentage selecting strongly disagree (2 versus 6%) compared to respondents with a household income of over NZ$100,000. A rural upbringing was also associated with the choice selection for this statement, with a higher percentage of respondents who grew up in rural areas agreeing (50 versus 45%) and fewer being neutral (4 versus 7%). Regarding the importance of pet insurance, age range, household income, qualification level, the presence of children in the household, and town or city dwelling were associated with the answer choices. For the age ranges 18–14 and 25–34, a higher percentage strongly agreed (22 and 25%, respectively), and a lower percentage selected neutral (34 and 35%, respectively) compared to all other age ranges up to 75 years (strongly agree range 0–13%; neutral range 49–61%). For household income, those with NZ$14,000–NZ$48,000 and NZ$48,000–NZ$70,000 had a lower percentage of respondents who selected ‘strongly agree’ (both 11%) compared with those with a household income of over NZ$100,000 (19%). A higher percentage of respondents with a level 7 qualification selected ‘strongly agree’ (19%) than those with a level 1 qualification (6%). For respondents with children, a lower percentage strongly agreed or agreed (11 and 19%, respectively, compared to respondents without children, 16 and 23%, respectively), and a higher percentage selected ‘disagree’ (18 versus 13%). The final statement in the finance section was, ‘People should only own a pet if they can afford to keep it.’ A higher percentage of those in the 18–24 and 25–34 age ranges strongly agree (67 and 66%, respectively) compared to those aged 55 to 64 (47%). For household income, those earning between NZ$14,000 and NZ$48,000 had a lower percentage of respondents who selected ‘strongly agree’ (48%) and a higher percentage selecting ‘neutral’ (15%), compared to those with a household income of over NZ$100,000 (63 and 7%, respectively). No other significant associations were observed.

Three binary logistic regression models were developed to identify the demographic factors predicting respondent agreement with the statements on the economics of pet care. The results of the analyses are summarised in Table 2 The final model for expense as a factor in pet care (χ^2^[4] = 22.983; *P* < 0.001) explained 2.3% of the variance and correctly classified 85.2% of cases. As shown in Table 2, higher household income was associated with decreased odds of agreeing that expense is a factor in veterinary care. Conversely, having more children, having a rural upbringing, or living in a town/city were all associated with increased odds of agreeing with the statement. The model for pet insurance, being important (χ^2^[5] = 162.779; *P* < 0.001) accounted for 12.2% of the variance with an overall classification accuracy of 68.2%. Increasing age and a higher number of children were associated with decreased odds of agreeing that pet insurance is important. In contrast, higher qualification level, identifying with a particular ethnicity (NZ European), and living in a town or city were associated with increased odds of agreement. The final model for only owning a pet if they can afford to be kept (χ^2^[3] = 37.834; *P* < 0.001) explained 4.2% of the variance and correctly classified 88.6% of cases. Higher household income was associated with increased odds of agreeing that people should only own a pet if they can afford it. Increasing age and a higher number of children were associated with decreased odds of agreeing with the statement.

**Table 2.** A summary of the forward stepwise (likelihood ratio) binary logistic regression analyses for 2019 New Zealand Pet Survey

Dependent Variable	Predictor Variable	B	SE	Wald	df	*P*-value	Odds Ratio
Expense is a factor (n = 1,754) Nagelkerke R^2^ = 0.023	Household Income	–0.159	0.056	7.984	1	0.005	0.853
	Number of Children	0.204	0.086	5.551	1	0.018	1.226
	Farm/Rural Upbringing	0.385	0.167	5.360	1	0.021	1.470
	Town/City	0.323	0.154	4.391	1	0.036	1.381
	Constant	1.904	0.258	54.266	1	<0.001	6.714
Pet insurance is important (n = 1,749) Nagelkerke R^2^ = 0.122	Age Range	–0.393	0.037	110.130	1	<0.001	0.675
	Number of Children	–0.293	0.064	20.829	1	<0.001	0.746
	Town/City	0.342	0.130	6.967	1	0.008	1.408
	Ethnicity	0.256	0.116	4.871	1	0.027	1.291
	Qualification Level	0.049	0.023	4.424	1	0.035	1.050
	Constant	–0.263	0.306	0.736	1	0.391	0.769
Financially capable (n = 1755) Nagelkerke R^2^ = 0.042	Household Income	0.294	0.061	23.032	1	<0.001	1.342
	Age Range	–0.195	0.049	15.642	1	<0.001	0.823
	Number of Children	–0.167	0.082	4.137	1	0.042	0.846
	Constant	1.822	0.272	44.744	1	<0.001	6.183

**Table 3.** Thematic analysis of comments related to some of the financial aspects of providing pet care among 2019 New Zealand Pet Survey respondents

Theme	Representative quotes
Vets and pet insurance are expensive	*“I think expense is a factor for many people because vets are very expensive.”* *“Pet insurance is too expensive.”* *“But not all vet care can be expected and planned for. And insurance is pricey. If you may not need … sad gamble…”*
Pet insurance exclusions	*“Pet insurance is unavailable for older cats – so my cat (age unknown) cannot get insurance.”* *“Pet insurance excludes a lot of conditions, especially if you own a large or giant breed dog.”*
Only have a pet if you can afford its basic care	*“If you cannot afford the basic care of a pet and all it entails, they shouldn’t get it.”* *“No matter how many benefits a person or family receive from having a pet cat or dog, if they are unable to afford it, the animal suffers.”*
Pets are a luxury/privilege	*“I feel that a pet is a luxury these days and people must see if their budget will allow for a pet before getting one.”* *“Owning a pet is a privilege.”* *“It’s a luxury not a right to have a pet.”*
Need to save	*“I put money aside at the vets on a regular basis. My form of health insurance.”* *“Pet insurance is expensive. We save money each week and use it to pay vets fees when required.”*
Financial situations change	*“…financial circumstances can change after a pet has been in the family for a while.”* *“People’s circumstances change they shouldn’t lose their pets because of this.”* *“Not everyone has the same circumstances, pets bring great joy to families who already may not have much, circumstances also change, important to be open-minded.”*
Assistance to pay	*“…people should have access to services that can help with the cost of pet guardianship – subsidised vet services, etc. To bar people on lower incomes is unfair.”* *“People fall on hard times sometimes – there should be support that includes them and their pets.”*
Pet care costs should be affordable	*“Vet care should be better regulated as costs are prohibitive. The fact that insurance is so highly recommended is indicative of this.”* *“Having a pet should be affordable.”* *“Children/families should have good/appropriate access to animals in their lives. Shouldn’t be prioritised by affordability.”* *“[There] should be options for people of limited means, e.g. pensioners.”*
Differing priorities	*“There are some people out there who don’t have much money but will always put their animals first.”* *“We can’t afford pet insurance, however, we would rather go without if it meant our cats needed medical attention.”* *“For some people, expense is a problem and pets go untreated if they cannot afford to pay.”*
Euthanasia	*“I do know that some dogs and cats get put down because that is the cheaper option sometimes for the owner which is sad.”* *“I hear of so many people who have their furbaby put to sleep just because they think the cost is too much. So sad.”* *“Veterinary clinics are basically a business and I have seen cases where people who cannot afford operations for their pets have had to have them euthanased, which is tragic.”*
Education and planning	*“… people will always take on pets not realising the costs – it’s a matter of education and have back up plans for people who get stuck (i.e. animal shelters agreeing to take on pets when people hit hard times, encouragement to set up saving schemes with the vet, advice on how to prevent vet bills in advance (e.g. getting your pet fixed).”*

## Discussion

The 2019 NZ Pet Survey found that financial constraints significantly impact pet guardianship, with factors such as age, household income, education level, geographic location, and pet insurance contributing to disparities in access to veterinary care. These findings mirror global research showing that cost can act as a barrier to low-income pet guardians accessing veterinary services, particularly preventive treatment (Morris *et al.* 2021; King *et al.* 2022). The current survey confirms this concern, highlighting how financial hardship affects not only the care pets receive but also the emotional well-being of their guardians.

Over 83% of respondents indicated that cost influenced the veterinary care their pets received — a concern that has likely intensified in the years since the survey due to worsening economic conditions in Aotearoa NZ, including rising inflation, housing stress, and the broader cost-of-living crisis (Wang *et al.* 2025). Similar to international studies (Spitznagel *et al.* 2019; Jatrana & Crampton 2020; Brown *et al.* 2021; King *et al.* 2022; Muldoon & Williams 2024a,b). These findings emphasise how financial hardship can hinder timely access to veterinary services, leading to poorer outcomes for animals and increased psychological stress for their caregivers.

Notably, rural respondents in the NZ survey were more likely to perceive financial constraints as a major factor influencing veterinary care decisions, suggesting disparities in access and cost structures between rural and urban settings. These findings align with international studies, which show that financial fragility is often worsened by limited geographic access to veterinary services due to the uneven distribution of veterinary staff (Morris *et al.* 2021). This highlights the urgent need for improved access to affordable veterinary care, particularly in rural communities.

Delays in treatment and inadequate access to preventive care were commonly associated with financial hardship. Several studies have documented that pet guardians experiencing financial stress may delay routine visits and long-term treatments (Chaumet *et al.* 2021; Forrest *et al.* 2023). As a result, these challenges increase emotional stress on pet guardians, who often view their inability to provide adequate care as a personal failure, further compounding their mental health burden (Spitznagel *et al.* 2019). Several responders reported that unexpected veterinary expenses, such as emergency surgeries, diagnostics, or ongoing management of chronic conditions, were common causes of stress for them. These findings are consistent with international studies showing that financial shock from emergency veterinary bills often leads to delayed care or economic euthanasia (Williams *et al.* 2020). Furthermore, participants in the current survey reported sacrificing other household necessities to meet pet-related costs, illustrating the broader impact of financial hardship on family well-being.

International solutions, such as financial planning tools, community support programmes, employer-backed pet insurance, and veterinary social work have been explored to alleviate these burdens (Kogan *et al.* 2021; Protopopova 2021). Educational interventions delivered during routine veterinary appointments may empower guardians to plan for future expenses. More accessible financial strategies — such as care packages or dedicated savings accounts — can provide realistic alternatives to conventional pet insurance. While insurance can offset costs and improve access to advanced care (Springer *et al.* 2022), it remains a limited solution. Only one-third of respondents considered it essential, noting that premiums are prohibitively high and that many policies exclude common conditions, particularly in older animals and certain breeds. This finding mirrors global trends of low uptake driven by factors such as perceived need, trust in insurers, and high cost (Brown *et al.* 2012; Paul & Skiba 2012; Chaumet *et al.* 2021). While pet insurance can mitigate financial barriers, its effectiveness is limited by high premiums and exclusions, particularly for older pets or specific breeds (Cunningham 2007; Williams *et al.* 2020; Becker *et al.* 2022). These findings highlight the need for greater transparency and inclusivity in insurance policies. Future research could explore regulatory strategies to broaden coverage, develop targeted insurance options for vulnerable groups, and improve public understanding of the risks and benefits associated with pet insurance (Chiu *et al.* 2021).

Some guardians described distressing decisions — including euthanasia — not based on medical advice but on affordability. These decisions caused long-lasting emotional impact. Veterinarians also experience moral injury when financial constraints prevent them from providing standard care (Kipperman *et al.* 2017). These findings highlight the need for systemic support, such as ethics training, communication frameworks, and psychological resilience programmes for veterinary professionals. The involvement of veterinary social workers or trained advocates could help guide clients through emotionally charged and financially constrained decisions (Quain *et al.* 2021). This study also highlights broader socioeconomic inequalities. Households earning under NZ$70,000 were significantly more likely to report financial stress and less likely to use pet insurance. These financial challenges were amplified among rural residents, families with children, and those with lower educational attainment. Beyond affordability, logistical barriers, such as limited transportation, clinic availability, and workforce shortages — especially in rural areas — further limit access to care (Morris *et al.* 2021; King *et al.* 2022; Muldoon & Williams 2024b). These intersecting challenges reveal systemic inequities that call for coordinated, multi-stage interventions. To address these disparities, public funding for essential veterinary services in underserved communities is a necessary step. International examples show that integrating animal welfare into existing social support systems — such as housing, aged care, and family services — can enhance both human and animal outcomes (Fox & Gee 2019; Adams *et al.* 2021).

Community-driven, preventive approaches were strongly supported by participants, many of whom recommended subsidised mobile clinics, savings education, and affordable care packages. These ideas mirror successful international initiatives aimed at improving access while reducing reliance upon insurance alone (Kogan *et al.* 2021). Equipping guardians with preventive care knowledge and cost-effective treatment options may increase preparedness and reduce stress. Policy-makers and veterinary associations should collaborate to create a supportive infrastructure to address financial barriers in pet healthcare. This could include tax incentives for veterinary donations, grants for community clinics, and incorporating veterinary services into social welfare assessments. Equally important is supporting veterinarians through training in financial communication, ethical decision-making, and burn-out prevention. Creating a national guideline for the ‘Spectrum of Care’ model would further enable veterinary professionals to tailor treatment options to a guardian’s financial capacity (Brown *et al.* 2021). This model improves access and supports business morale by legalising the financially constrained care routes.

These recommendations also align with broader social goals. Promoting animal health contributes to the well-being of families and communities, echoing the objectives of SDG 3 (Good Health and Well-being). By analysing how income, education, and geography affect access to veterinary care, this research also supports SDG 10 (Reduced Inequalities) and SDG 11 (Sustainable Cities and Communities), particularly through its call for more inclusive, pet-friendly policies and urban planning.

Given the scale and complexity of financial challenges, future research should examine the behavioural psychology of pet care, including how pet-related expenses are prioritised in household budgets. Qualitative studies capturing the lived experiences of economically diverse groups — especially Māori and Pasifika communities — are needed to inform culturally appropriate and equitable policy (Forrest *et al.* 2023).

In addition, the COVID-19 pandemic further exacerbated these challenges, amplifying the financial strain on pet guardians and highlighting the interconnectedness of human and animal health (Enders-Slegers & Hediger 2019; Applebaum *et al.* 2020; Bibbo *et al.* 2022; Charmaraman *et al.* 2022; Meier & Maurer 2022). This has highlighted the importance of integrating animal welfare considerations into future public health emergency preparedness and response efforts, as advocated by the One Health Framework (Adams *et al.* 2021; Protopopova 2021). By identifying systemic barriers and proposing integrated solutions, this research contributes to a more equitable and resilient model of companion animal care — one that acknowledges the shared well-being of people and their animals.

### Study limitations

While offering insights into the financial aspects of pet guardians, this study acknowledges certain limitations. The voluntary nature of the online survey led to self-selection bias, resulting in a non-representative sample with a disproportionately high number of non-Māori individuals and females compared to the national population. Furthermore, the data were collected in early 2019, over six years ago and before the COVID-19 pandemic. Given the significant economic disruptions caused by the pandemic and the worsening cost-of-living crisis since then, the findings may not fully reflect the current realities faced by NZ pet guardians. However, the findings provide a valuable pre-pandemic baseline for future research on how financial and social conditions have influenced attitudes and practices over time.

### Animal welfare implications

Overall, the findings of this study highlight both the direct and indirect impacts of financial constraints on animal welfare and the well-being of their guardians in Aotearoa NZ — impacts that are increasingly relevant in the current economic climate. Since the survey was conducted in 2019, NZ has experienced significant economic challenges, including rising inflation, increased cost of living, and worsening housing affordability. These shifts have likely intensified the financial pressures already reported by participants, further limiting their ability to access preventive care or timely treatment for their animals.

The study suggests that when pet guardians are unable to afford necessary treatment and preventive care, it often results in delays that can lead to worsening health conditions or, in some cases, euthanasia. Additionally, the emotional stress experienced by guardians who are unable to provide appropriate care for their pets can negatively affect their decision-making, further compromising the welfare of the animals in their care. Given the ongoing economic pressures, it is likely that these issues are now even more widespread and urgent than they were in 2019.

## Conclusion

The financial aspects of pet guardianship present a complex challenge, impacting both the well-being of the pet and its owner. This study, along with a growing body of global research, highlights the need for comprehensive strategies to promote sustainable and equitable pet guardianship. Policy-makers, veterinary professionals, and animal welfare organisations must collaborate to develop innovative solutions that improve access to affordable veterinary care, enhance financial literacy among pet guardians, and provide support systems for those facing economic hardship. By addressing these challenges, we can foster a world where the human-animal bond thrives, promoting the health and happiness of both pets and their guardians in alignment with the One Health Framework and the Sustainable Development Goals.
